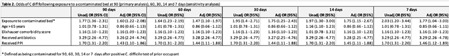# Impact of exposure to potentially contaminated hospital beds on risk of hospital-onset C. difficile infection

**DOI:** 10.1017/ash.2022.86

**Published:** 2022-05-16

**Authors:** Lucy Witt, Jessica Howard-Anderson, Elizabeth Overton, Jesse Jacob

## Abstract

**Background:** Environmental contamination increases risk for *Clostridioides difficile* infection (CDI) given that spores can remain on a hospital bed, floor, sink, and light switch despite appropriate cleaning measures. Using real-time asset management software (AgileTrac, GE Healthcare) for beds we examined the risk of a patient developing hospital-onset CDI (HO-CDI) when staying in a hospital bed that had a previous occupant with CDI. **Methods:** We retrospectively identified all patients in tracked beds from April 2018 to August 2019 to identify hospital-onset CDI (HO-CDI), defined as a positive PCR test for *C. difficile* in a patient hospitalized for >3 days. A patient was defined as being exposed to a potentially “contaminated” bed if within the preceding 7 days from their HO-CDI diagnosis they resided in a hospital bed that, within the prior 90 days, had held an occupant with CDI (Fig. 1). We used multivariable logistic regression to evaluate the association between being exposed to a contaminated bed and HO-CDI. Model covariates were chosen a priori based on known risk factors for CDI. As a sensitivity analysis, we varied the length of time that a bed could stay contaminated from 90 to 60, 30, 14, and 7 days. **Results:** We analyzed 25,032 hospital encounters representing 18,860 unique patients; we identified 237 (0.9%) hospital encounters with HO-CDI (Table 1). The Elixhauser comorbidity score, being exposed to a contaminated bed, and receiving antibiotics or a proton pump inhibitor (PPI) during the hospital admission were all associated with HO-CDI in the univariable analysis (Table 2). In the adjusted multivariable model, being exposed to a contaminated bed remained a significant risk factor for HO-CDI (OR, 1.60; 95% CI, 1.22–2.08) even after controlling for known risk factors for CDI including age >65, elevated Elixhauser score, and recent antibiotic or PPI use (Table 2). In the sensitivity analysis in which we adjusted the time a bed was considered contaminated after CDI, being exposed to a contaminated bed remained a risk factor for HO-CDI, with a similar odds ratios as the original model (Table 2). **Conclusions:** Residing in a hospital bed that contained a previous occupant with CDI is a risk factor for developing HO-CDI. Hospital epidemiologists, infection control personnel, and environmental services staff should consider this association when developing CDI risk mitigation strategies.

**Funding:** None

**Disclosures:** None

**Figure:**
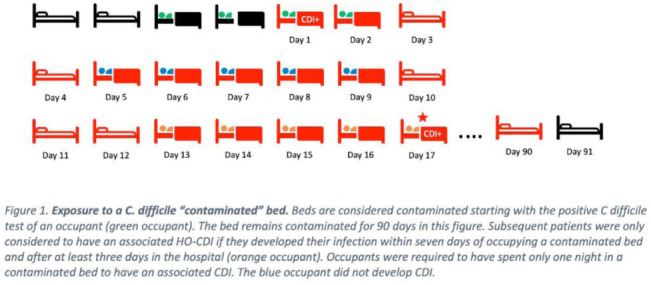

**Table:**
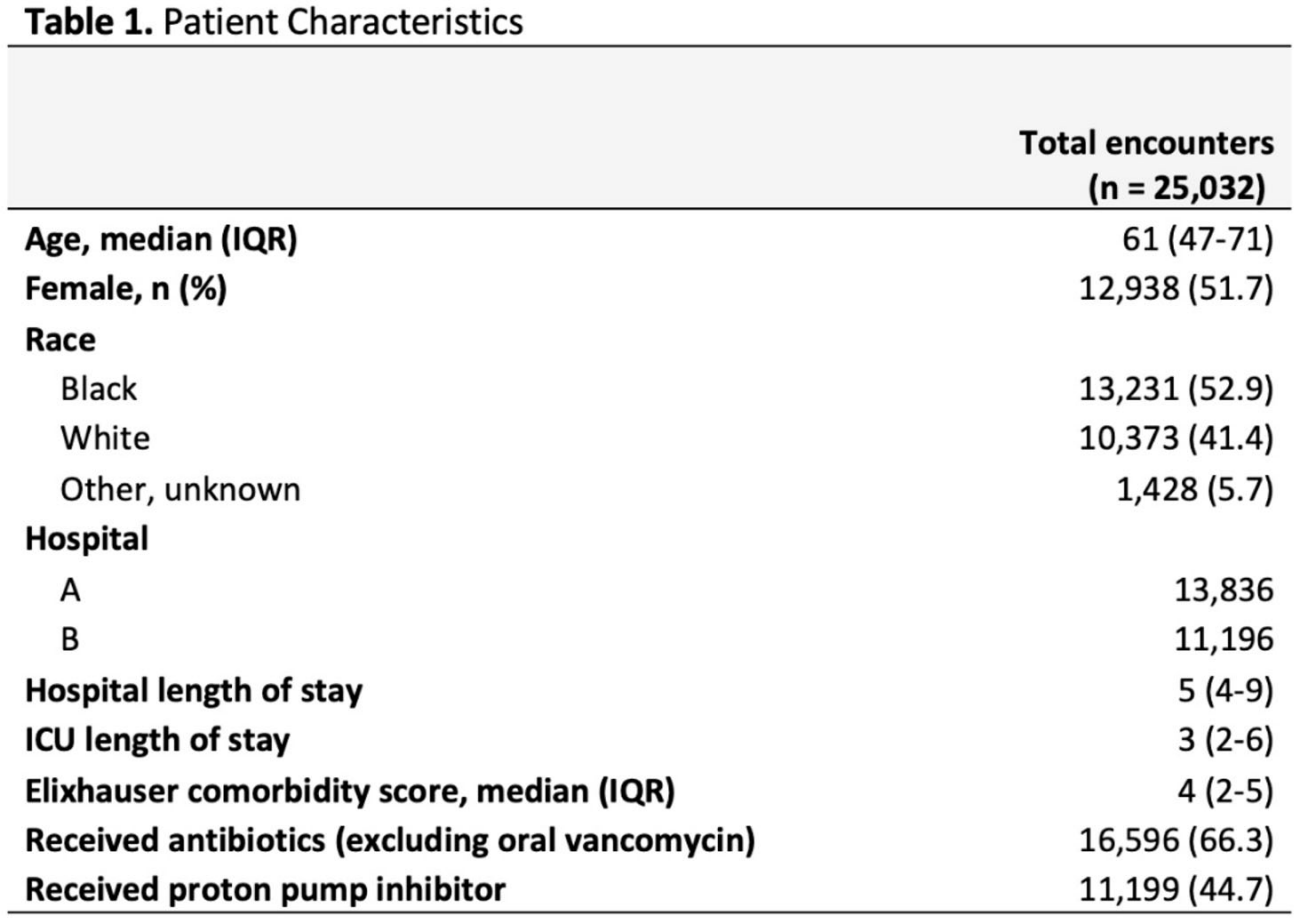

**Table:**